# App icon similarity and its impact on visual search efficiency on mobile touch devices

**DOI:** 10.1186/s41235-018-0133-4

**Published:** 2018-10-17

**Authors:** Anna K. Trapp, Carolin Wienrich

**Affiliations:** 1 Department of Psychology and Ergonomics, Technische Universität Berlin, Marchstr. 23 – MAR 3-2, 10587 Berlin, Germany; 2 Department of Human-Computer-Media, Julius-Maximilians-Universität Würzburg, Am Hubland, Campus Nord, Oswald-Külpe-Weg, 82, 97074 Würzburg, Germany

**Keywords:** Mobile touch devices, Visual search, Icons, App icons, grouping, Similarity manipulation, User experience, Attentional guidance

## Abstract

Users of mobile touch devices are often confronted with a great number of apps, challenging an efficient access to single applications. Especially when looking for infrequently used apps, users have to perform a visual search. We address this problem in two studies by applying knowledge about visual search efficiency to app icons on mobile touch devices. We aimed to transfer findings of similarity grouping for complex stimuli to a more applied setting and to investigate the effect of search efficiency on user experience. In Study 1 (*N* = 18), we varied set size and target presence as well as visual similarity between icons by color manipulation. Results indicated a highly efficient search when the target was easy to discriminate from the distractors and a less efficient search with increasing similarity. These results were replicated in a second, more realistic use case (*N* = 36). Regarding user experience, Study 2 showed that perceived usability and intuitiveness increased with search efficiency but that the overall liking also depended on the visual variety of the design. Moreover, although participants showed a general interest in a system supporting their search, most participants had concerns about data privacy with such a system. In conclusion, the results indicate that concepts and findings from basic attention research serve as fruitful heuristics for searches in more realistic (applied) settings. Furthermore, results showed that similarity manipulation with color works without controlling for other icon characteristics (e.g. luminance, shade). The findings might offer a new approach when designing for smooth interaction with mobile touch devices.

## Significance

When users look at their mobile touch device to open an app, they first have to find the right icon. We investigate whether searches can be supported by colored app icons and how users feel about such a support system. It is known that search efficiency can be manipulated by similarity grouping. However, these results are mainly based on simple stimuli and a yes/no answer format. App icons are more complex because they have many different visual features and users have to tap the icon to start the app after navigating between different screens of the mobile device. This paper aims to transfer basic knowledge from visual search for these complex stimuli by applying the theoretical foundation of visual search in the context of mobile touch devices. This includes not only similarity grouping, effects of set size, and target presence, but also motoric processes. Additionally, we aim to connect search efficiency with user experience (UX). The results give a first insight into how icons can be designed to allow grouping, which can be used in further research regarding spatial learning processes, semantic categorization of apps, and individual preferences of app organization. The results indicate how important the overall design of mobile device screens is for UX. Moreover, a color grouping scheme might be a way to further empower designers to develop devices that match the user’s hedonistic and pragmatic needs.

## Background

On mobile touch devices, efficiency refers to quick access to apps with a minimum of resources spent on the search. However, the increasing number of available apps forces users into a complex and inefficient visual search task. The complex search dynamic arises from factors such as the available screen size of mobile devices, similarity of app icons, the need to swipe through several smartphone pages, diverse use-environments (e.g. on a train, while walking), differing goals and use cases leading to altering target apps, and the need for complex motoric responses (e.g. touch displays with little or no haptic feedback). The search for an app icon is even further complicated because phone manufacturers often implement the possibility to adjust (“individualize”) the spatial icon array. Even though personalization can allow quicker access to certain apps, most phone manufacturers limit the degrees of freedom offered to arrange app icons and personalized arrangements can be altered by updates. Hence, human-centered app icon design faces the challenge of facilitating the complex visual search task on mobile touch devices. Our goal was to transfer knowledge from basic research on visual search to the field of human–computer interaction and app icon design, with the aim of providing mobile touch device users with a fluent experience when searching for an app. We first examined visual search efficiency for app icon selection by similarity grouping with universally applicable colored icons (Study 1). Visual search efficiency was then further investigated with regard to the appeal of these colored icons and their effect on the perception of the interaction qualities in terms of UX (Study 2). Three theoretical areas were considered: basic research regarding visual search; applied research dealing with visual search of icons; and the impact of (search) efficiency on UX.

### Guidance of attention in visual search: Basic research

#### Artificial visual search

In basic experiments, visual search requires the scanning of spatial locations to identify a target item among a field of one or more distractor items. The reaction time as function of the number of items is often referred to as search rate or slope. It is a measure of the search efficiency (Treisman & Gelade, 1980). Low search rates show a more efficient search than high search rates (see Wolfe, 1996, for a review). The search rate is highly correlated with the target’s saliency, which results from physical attributes of the stimuli (i.e. bottom-up), as well as from the amount of selective attention paid to these contrasts (i.e. top-down) (see Moran & Desimone, 1985; Kastner & Ungerleider, 2000). Theoretical models of visual search (e.g. Guided Search Model; Cave & Wolfe, 1990; Wolfe, 1994) assume that attention is driven by an overall (master) map representation of integrated—bottom-up and top-down—priority signals whose output lead to a continuum of visual search results.

The Guided Search Model discriminates between a pre-attentive and an attentive stage. The pre-attentive stage determines the weights of the values represented on the saliency map. The saliency map corresponds to the space in the visual field. Its values correspond to the significance of the visual information at the matching locations (Findlay & Gilchrist, 1998). At the attentive stage, attention is gradually guided to the highest points on the saliency map. Hence, the Guided Search Model describes how basic features (e.g. color) as well as internal states of the observer (e.g. goals) guide the deployment of attention via bottom-up and top-down processes. Many behavioral and neuroscientific investigations have confirmed the operation of these bottom-up and top-down processes in the guidance of attention in a variety of search tasks (e.g. Gaspar & McDonald, 2014; Geng, DiQuattro, & Helm, 2017; for reviews see Fecteau & Munoz, 2006 or Wolfe & Horowitz, 2017). But what makes guidance more or less efficient?

#### Guidance by similarity

**Fig. 1:**
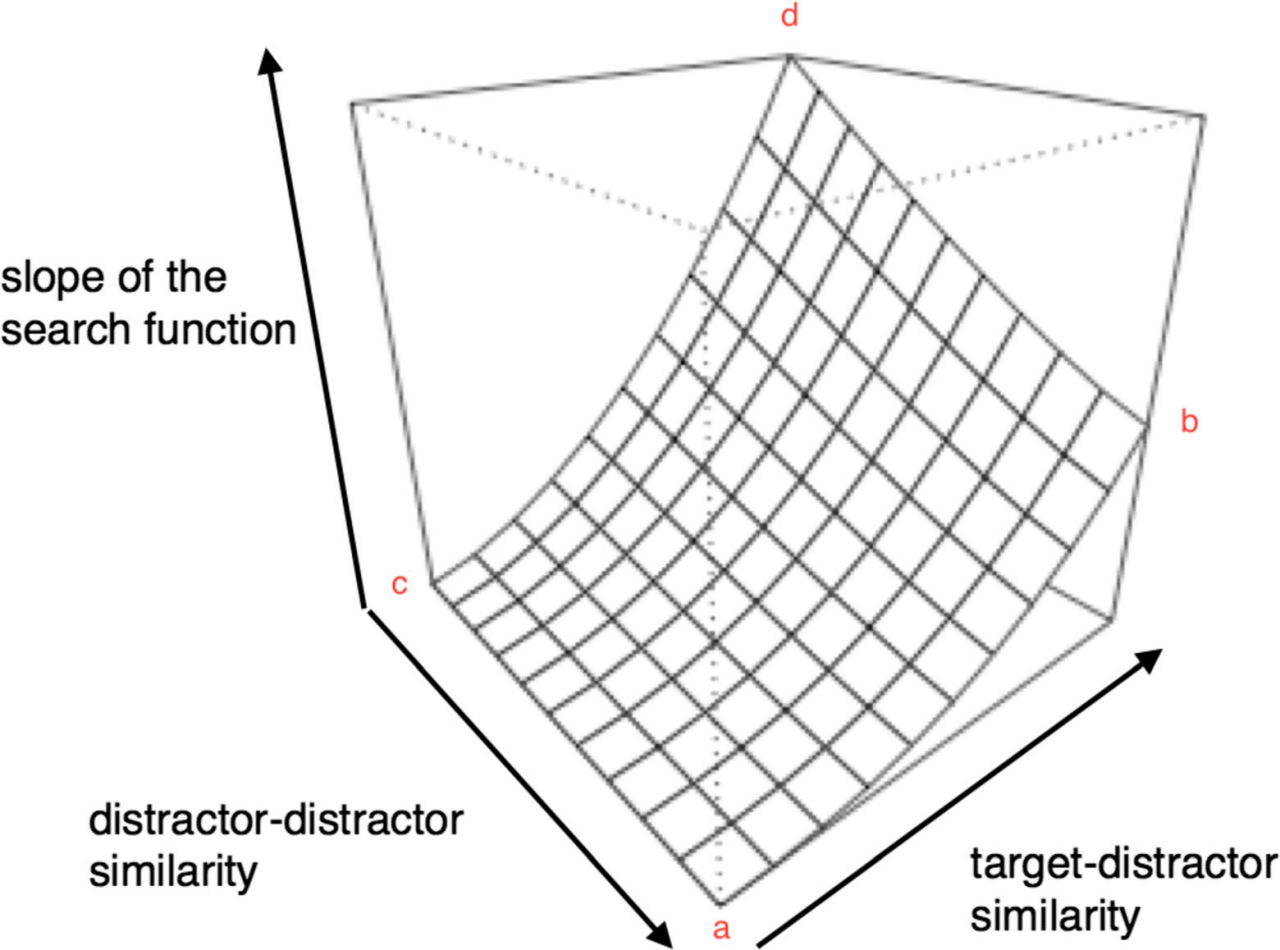
The search surface. Slope of the search function indicates the slope of reaction time over an increasing set size. It is plotted as a function of target–distractor similarity (TDS) and distractor–distractor similarity (DDS). Due to the grouping of similar items, high search efficiency arises in conditions with low TDS (points a and c). Here, the target visually “pops out.” Low search efficiency arises on the opposite corners of the cube. It is especially pronounced when DDS is low (point d). Adapted from Duncan & Humphreys (1989, p. 442)

Similarity strongly modulates the salience of a target. In the Attentional Engagement Theory, Duncan and Humphreys (1989) argued that different combinations of similarity relations between display items model the search efficiency, based on a visual grouping effect. More precisely, they describe a pre-attentive stage in which perceptually similar items (e.g. as indicated by similar orientation) form groups which can be jointly rejected or accepted for the selection process. Hence, the saliency of a target, and thereby the search efficiency, decreases with increasing target–distractor similarity (TDS) or with decreasing distractor–distractor similarity (DDS) (see Fig. 1). In the past, a wide range of similarity manipulation has been tested with regard to effective grouping (e.g. using letters: Corcoran & Jackson, 1977; using lines: Treisman & Gormican, 1988; using color patches: Farmer & Taylor, 1980). A key finding is that similarity differs along simple dimensions such as color and orientation (e.g. using orientation: Duncan & Humphreys, 1989; using color: Duncan, 1989; Bundesen & Pedersen, 1983).

#### Lessons from these models

Saliency is determined by bottom-up features (e.g. color contrast) and top-down factors (e.g. the relevance of color to the task). It guides attention during visual search. Moreover, target saliency can result from similarity relations, e.g. a red bird (target) in front of green leaves (distractors) will be highly salient because the target is dissimilar from the distractor while the distractors are highly similar to each other. In other words, the Guided Search Model (based on saliency) and the Attentional Engagement Theory (based on similarity relations) predict similar results for many search conditions. However, their predictions differ when comparing points b and d of the search continuum (see Fig. 1). Due to the fact that the Guided Search Model predicts a decreasing search efficiency as more items show the same characteristic in a task-relevant feature (e.g. red fruit in a search for red apples), steeper reaction time slopes are assumed for corner b than for corner d in Fig. 1. The opposite is predicted by Attentional Engagement Theory, according to which search rates increase with decreasing DDS and thus the requirement of the rejection of an increasing number of heterogeneously colored distractor items/groups in the process of visual search. We aim to transfer the knowledge from the above-mentioned basic research to the applied setting of visual search on mobile touch devices and to test the two predictions against each other. Hence, with the help of colored app icons, the predictions of the models were tested for four extreme points (compare corners a, c, b, and d of Fig. 1) of the visual search continuum.

### Guidance of attention on mobile devices: Applied research

Well perceivable display shapes (e.g. through well-defined perceptual groups) also enable a fluent search (see, for example, Scott, 1993 for a detailed review) under more realistic search environments using app icons as search elements. McDougald and Wogalter (2014) focused on the influence of color to guide the user’s attention. They reported more correct descriptions of pictograms if relevant areas of the pictograms were highlighted by color than if there were no highlighting. The authors concluded that color directs the user’s attention to relevant areas and consequently facilitates comprehension. However, the impact of color-highlighting was only shown in terms of correct answers rather than search time. Hence, the effect on visual search efficiency could not be elucidated. In another study, participants were asked to rank colored icons according to their noticeability (Bzostek & Wogalter, 1999). The authors presented warnings at different screen locations, using different icons and colors. In line with the subjective perceptions, participants were able to notice warnings faster if they were presented with colored icons (blue and red) in comparison to black icons within a black inked text. Other factors such as icon location yielded no additional benefit in the color-present conditions. Results of both studies indicate that color effectively guided attention to relevant areas of the screen.

Other studies have shown that grouping of icons facilitates visual search. Niemelä and Saarinen (2000) found a more efficient search for spatially grouped icons compared to non-grouped icons or non-icon-items (words) on a computer screen. They presented 16 icons with file names in a 4 × 4 grid. Due to arranging four items spatially close together, these items formed perceptional groups that facilitated the search in comparison to random arrangements. Likewise, Brumby and Zhuang (2015) found a facilitation of visual search in menu interfaces due to semantic order and visual grouping, depending on the group size. Semantic order was implemented by listing words of one category together. Visual grouping was realized by framing words of one category. Visual grouping was more effective for larger semantically organized groups (six icons) than for smaller ones (three icons). They also showed the importance of semantic and visual accordance. Visual grouping of semantically unrelated icons was significantly slower in comparison to no grouping. Both studies showed that visual search on a computer screen can benefit from spatial grouping in certain conditions (e.g. semantic and visual accordance). However, neither a mobile nor a touch device was tested.

In sum, we know from applied research that guidance by simple features (e.g. color) and similarity grouping modulates the efficiency of visual search. Notwithstanding the evidence that color highlighting and similarity grouping are important factors of app arrangement on mobile touch devices, a systematic transfer of fundamental visual search results to this applied setting has not been done yet. A validation of results from visual search paradigms in the applied context of mobile touch devices might significantly contribute to the design of more efficient interfaces. Here, we also aim to test how users feel about applications modifying their search behavior. This aim is based on the user-centered design approach, which emphasizes the importance of including the user in the process of designing technology (ISO 9241–210, 2010; Norman & Draper, 1986).

### User experience and the efficiency of use

The ISO standard on the ergonomics of human system interaction defines UX as “a person’s perceptions and responses that result from the use or anticipated use of a product, system or service” (ISO 9241–210, 2010, p. 7). Based on this definition, the components of the UX model (CUE model, Thüring & Mahlke, 2007; Minge, Thüring, Wagner, & Kuhr, 2016) assumes three major components of UX: the perception of instrumental qualities; the perception of non-instrumental qualities; and the experienced emotions. Instrumental qualities describe attributes of the system that are beneficial for the task such as usability and usefulness of the product. Non-instrumental qualities are not essential for completing the task and refer to aspects of the visual attractiveness, aesthetics, and of the increase of one’s own status. Emotions describe the inner state of the user that is affected by the interaction. All three components affect the consequences of the interaction with a product, such as the global UX evaluation and acceptance of the system, or the intention of reuse. Conversely, the components are also influenced by the user, the design of the product, and the context of the use. A wide range of studies found results supporting the framework of the CUE model (Aranyi & van Schaik, 2015; Ben-Bassat, Meyer, & Tractinsky, 2006; Hamborg, Hülsmann, & Kaspar, 2014; Lee & Koubek, 2012; Mahlke, Minge, & Thüring, 2006; Minge & Thüring, 2018; Thüring & Mahlke, 2007). These studies show that usability is understood as one of the key components for the perception of instrumental qualities, while visual attractiveness is one of the key components in the perception of non-instrumental qualities.

Focusing on usability, a connection between efficiency and UX appears. As usability is defined as the extent to which a product or service can be used effectively and efficiently while being satisfying to the user (ISO 9241–210, 2010), it becomes clear that efficiency of use is a major component of usability. In fact, efficiency measures in terms of temporal units per successfully completed task are frequently used in order to infer the objective usability of a device (Agarwal & Meyer, 2009; ISO 9241–210, 2010; Hamborg et al., 2014; Mahlke, 2008) and its intuitiveness (Blackler, Popovic, & Mahar, 2003; Blackler, Popovic, & Mahar, 2010). The more efficiently an interaction can be accomplished, the higher the objective usability and the intuitiveness of that device are. Moreover, efficiency measures correspond to reaction times in the visual search paradigm, as they are also a temporal measure per correct response. Hence, reaction time measures are comparable to “total task time”—or “time on task”—measures that are frequently employed in usability and UX testing. Furthermore, they could also be seen as an indicator of the efficiency of the interaction and not only of the efficiency of the search itself.

In sum, in an applied visual search paradigm on mobile touch devices, the efficiency of the search can be understood as an objective indicator of the usability. The efficiency, on the other hand, is also perceived by the user, which influences the perception of the intuitiveness and of the instrumental qualities of the device. Due to the fact that the present paper aims to optimize search screens on a mobile touch device, an increased efficiency should be reflected in a better UX and increased intuitiveness.

### Aim of the present paper

Our goal was to increase the efficiency of app selection on mobile touch devices by using an organization scheme of app icons based on color. More precisely, we investigated whether basic findings from the visual search paradigm also hold for a more complex search situation on app icons and how users experience the colored icon, which presumably helps to improve their search. In order to realize the first step of transfer, an artificial visual search with real app icons on a mobile touch device was conducted in Study 1, aiming to replicate effects of TDS as well as DDS. Reaction times were analyzed depending on of set size and target presence. In Study 2, the effect of the TDS and DDS was replicated and its impact on UX and intuitiveness was investigated. Additionally, further aspects of the transformation of basic research to the applied setting of app search on mobile touch devices, such as response format (touching the target) and swiping, were realized. Finally, other important impacts, such as crowding (Pelli & Tillman, 2008) or guidance by memory (e.g. Chun & Jiang, 1998; Geyer, Zehetleitner, & Müller, 2010) were discussed as directions for future work.

## Study 1

### Methods

#### Participants

Eighteen participants (11 men) with a mean age of *M* = 25.6 (*SD* = 2.7) years took part in the first study. The sample size was computed with help of the pwr-package for R (Champely, 2018) and adjusted upward to fit the balancing process, expected *d* = 1, *α-level* = 0.05, *power 1-β* = 0.8. All participants had normal or corrected to normal vision, with no instances of color-blindness. Fifteen 15 of the participants owned a smartphone. Participation was voluntary. Participants were offered course credits for participating. Before the beginning of the experiment, participants gave their informed consent according to the WMA Declaration of Helsinki.

#### Stimuli and design

**Fig. 2:**
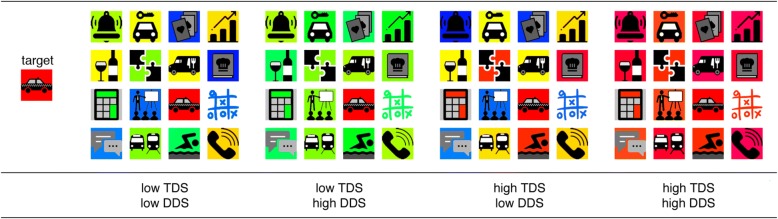
An example of search screens in the four similarity conditions (target present). The figure shows how TDS and DDS were manipulated to vary similarity between the icons. These are example icons that are highly similar to the icons used in Study 2. Commercial icons from the first study cannot be presented due to copyright protection

A total of 26 icons were used; 25 of these were retrieved from the Apple App Store or from Google Play and one was designed by the authors. All icons measured 2.5 × 2.5 cm. There was a gap of 3 mm between icons. Participants sat at a desk. The test device lay on the desk. Participants were able to move freely while seated.^1^ None of the icons included recognizable letters or numbers and all icons were transformed into a flat design (i.e. no color gradients) and set to black and white. Color was added to vary TDS as well as DDS between icons. To this end, 20 colors were chosen from the HSV color model. Each color belonged to one of four color groups (red, blue, yellow, and green). The colors differed in terms of their hue (red: 340°, 350°, 0°, 10°, 16°; yellow: 50°, 53°, 56°, 59°, 62°; green: 80°, 90°, 100°, 125°, 140°; blue: 210°, 216°, 222°, 228°, 234°), while saturation and value (in terms of lightness) were kept constant at 100%. The colors were chosen to provide high discriminability between color groups and high similarity within color groups. However, we ensured that the colors within one color group were still distinguishable. All icons were colored in each of the 20 colors, resulting in 520 colored icons. The icons differed in their overall luminance as well as the percentage of colored area as real-life app icons do. Colors for distractor icons were chosen to originate either from the same color group as the target (high TDS) or from another color group (low TDS) (see Fig. 2). Distractor colors were chosen from the same color group (high DDS) or from three different color groups (for low DDS). Targets were always presented in a unique color (0°, 222°, 56°, or 100°) while distractor colors were chosen from the other 16 possible colors. In accordance to smartphone displays, *set size* was varied between the level 8, 16, and 24 icons. The target was present in half of the trials (*target presence*). In sum, the study was based on a four-factorial within-design (3 × 2 × 2 × 2) with the factors set size, target presence, TDS, and DDS.

#### Apparatus

The study was conducted on a 10-in. resistive touch device (Faytech) with a resolution of 1024 × 768, connected to a PC running ePrime (version 2.0).

#### Procedure

Trials started with a fixation cross in the middle of the screen. Participants were instructed to hold their forefinger over the fixation cross and tap the screen when they were ready to start. Once tapped, a colored target icon was presented for 2 s followed by another fixation cross. Again, participants had to tap to show they were ready before the search screen was presented. Each search screen consisted of 8, 16, or 24 colored icons, arranged in rows of four, and one gray box at the bottom of the screen. Participants were instructed to tap the target or to tap the gray box if the target was absent. After the response, a feedback screen was shown with information regarding the correctness of the response, the response time (in milliseconds) and how well they were performing during the ongoing block (percentage of correct trials).

The experiment was divided into 12 blocks according to set size, TDS, and DDS. Blocks with the same set size were presented consecutively while the order of set size presentation was balanced over participants. For each set size, the order of the four similarity conditions was randomized. Each block consisted of 24 randomized trials, of which 12 were target-present and 12 were target-absent trials. The total number of trials was 288 per subject (12 per condition). Before starting the main experiment, participants performed a short tutorial to become acquainted with the experimental procedure.

Of the 26 icons, no icon was shown twice as a target in one block. However, an icon did reappear as a distractor. Furthermore, no icon was presented twice on a search screen. The target color (red, blue, yellow, or green), color groups, and target position were balanced for each participant.

#### Data analysis

The data analysis was conducted with R version 3.3.2 (R Core Team, 2016) and the following packages: afex (Singmann, Bolker, Westfall, & Aust, 2016); car (Fox & Weisberg, 2011); dplyr (Wickham & Francois, 2016); ez (Lawrence, 2016); ggplot2 (Wickham, 2009); MASS (Venables & Ripley, 2002); and tidyr (Wickham, 2016). Before the data analysis, all incorrectly answered trials (4.78%) were excluded from the dataset and reaction time data were transformed using a Box-Cox power transformation ( λ= − 0.55) to correct for positive skew (Venables & Ripley, 2002). The formula

$$ R{T}_{transformed}=\frac{R{T}^{\lambda }-1}{\lambda } $$

was used to compute transformed reaction time (*RT*_*transformed*_) from reaction time (*RT*) with *λ* as an exponent in the exponential transformation. This power transformation is based on a log-likelihood estimation for an optimal *λ*. With this procedure, the order of the original data is retained. Thus, high or low values in reaction time translate into high or low values in the transformed reaction time, respectively. Subsequently, an outlier analysis was conducted to exclude trials with reaction times differing by more than two standard deviations from the mean, which was calculated separately for each participant and condition.^2^ The remaining data (92.07%) were used to calculate slopes over set size for each participant and condition based on the variables TDS, DDS, and target presence. Hence, for each participant, there were eight slopes indicating the increase of reaction time with increasing set size. Due to the Box-Cox power transformation, these slope gradients were normally distributed. Subsequent analyses were based on slope values. All presented error bars were corrected for within-subject variance by the method suggested by Cousineau (2005). The alpha level was set at 5%. All post-hoc t-tests were corrected by Bonferroni–Holm correction.

### Results

The statistical analysis was conducted with an ANOVA (type III) on slope values with the independent within factors TDS, DDS, and target presence. The results are presented in Table 1.

**Table 1:** ANOVA (type III) on slopes over set size

	F	dfs	*p*	ges
Intercept	183.76	1, 17	< 0.001	0.84
TDS	142.59	1, 17	< 0.001	0.46
DDS	1.02	1, 17	0.328	0.00
Target presence	8.09	1, 17	< 0.05	0.07
TDS: DDS	39.76	1, 17	< 0.001	0.13
TDS: target presence	39.23	1, 17	< 0.001	0.08
DDS: target presence	1.61	1, 17	0.222	0.01
TDS: DDS: target presence	1.00	1, 17	0.331	0.00

Effects of target–distractor similarity (TDS), distractor–distractor similarity (DDS), and target presence on slopes over set size. The slopes were computed based on the transformed reaction time. Generalized eta square (ges) was computed as the effect size. Based on Bakeman (2005) a ges of 0.02 can be seen as a small effect, one of 0.13 as a medium effect, and one of 0.26 as a large effect

**Fig. 3:**
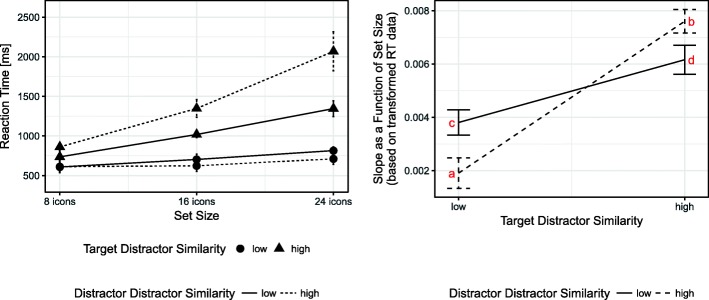
Interactions of TDS and DDS on reaction time and on slope values as a function of set size. Small slope values indicate a search that is mostly independent of set size, i.e. is very efficient. Large slope values suggest inefficient search processes. In order to compare the results to the predictions of Duncan and Humphreys (1989), depicted in Fig. 1, the letters a to d were added to the *graph* on the *right*. All analyses were conducted on slope values based on transformed reaction time (*right graph*), although original values are also shown (*left graph*). The slope values based on the original data for low TDS condition are 13 ms/icon (low DDS) and 6 ms/icon (high DDS) and for the high TDS condition are 38 ms/icon (low DDS) and 75 ms/icon (high DDS)

The results revealed a large main effect of TDS. As expected, the increase of reaction time over set size was much steeper (i.e. increasing slopes) when TDS was high than when it was low. DDS showed no significant impact on slope gradients. However, the expected interaction between TDS and DDS was present and revealed a medium effect size. The interaction is visualized in Fig. 3. The graph on the right shows that slopes were smaller when TDS was low (points a and c) compared to high TDS (points b and d). However, the slopes differed significantly between DDS levels at both TDS levels. When TDS was low, slope values were significantly smaller with high DDS compared to low DDS (points a and c, respectively), *t*(17) = 5.15, *p* < 0.001, *d* = 1.23. Conversely, when TDS was high, slope values were larger with high DDS compared to low DDS (points b and d, respectively), *t*(17) = − 4.39, *p* < 0.001, *d* = 1.05.

Regarding target presence, the ANOVA revealed a small effect: slopes were steeper if the target was absent. As expected, this effect interacted with TDS, target presence had no effect when TDS was low, *t*(17) = − 0.27, *p* = 0.787, *d* = 0.07, but a strong effect when TDS was high, *t*(17) = 5.25, *p* < 0.001, *d* = 1.24. This is in line with expectations and suggests once again an efficient search process when TDS is low and an inefficient process when TDS is high.

We reran the analysis with only those individuals who owned a smartphone. Within this subgroup, the main effect of target presence was only marginally significant and the interaction between TDS and DDS was more pronounced. However, the overall results were in line with the results of the entire group.

### Discussion

Study 1 aimed at transferring effects of TDS as well as DDS to app icons on mobile touch devices. For this purpose, reaction times were analyzed depending on similarity (between colored app icons), set size (number of app icons), and target app presence. As expected, grouping of similar app icons occurred and enabled an efficient app selection. Hence, our results mirror those from basic (laboratory) visual search studies. Regarding the different predictions of the Guided Search Model and the Attentional Engagement Theory, the results support the prediction of the Guided Search Model (compare corner b and d in Fig. 3 to Fig. 1). When the target is highly similar to the distractors (high TDS), the Guided Search Model predicts a weaker guidance of attention by a basic attribute (e.g. color), the more distractors carry this attribute. In the same scenario, the Attentional Engagement Theory predicts more efficient grouping and rejection with increasing distractor similarity (i.e. leading to steeper reaction time slopes for corner d than b in Fig. 1). When realizing point b, we created an extreme variant of high TDS and high DDS in which all icons shared the same color group. Thus, grouping by color could not offer any relevant information for target–distractor discrimination. Thus, the present pattern of results is not fully compatible with the prediction of Attention Engagement Theory. Instead, the results suggest that priority map models of the search process such as Guided Search are more appropriate to predict search performance in app icon search.

In accordance with basic research findings, interaction effects of TDS and target presence revealed characteristic patterns of efficient and inefficient search processes. With regard to the more applied line of research, our findings are in line with previous research recognizing the influence of color highlighting of computer pictograms (e.g. Bzostek & Wogalter, 1999; McDougald & Wogalter, 2014) or emphasizing the impact of grouping of icons (e.g. Brumby & Zhuang, 2015; Niemelä & Saarinen, 2000).

Although basic research results were confirmed for a mobile touch device setting, a major limitation is that the visual search on mobile touch devices was not adequately replicated. In real life, users mentally visualize the app icon and its features before a search. The location and color of icons are learned and memorized through the interaction with the device. In Study 1, these two dimensions were excluded by randomization in order to focus on effects of similarity grouping. The impact of spatial learning and memory, the preparation of the search by learned colors, as well as the interplay of these complex cognitive processes are further addressed in the general discussion.

Furthermore, icons on mobile touch devices are frequently presented on multiple screens and the user might have to swipe through several screens to find the desired app. That scenario implies an investigation of similarity grouping and learning across multiple screens. Finally, hedonic aspects of color organization schemes and of an accelerated search were not considered in Study 1. Thus, it seemed doubtful whether the increased efficiency of a visual search will be accompanied by a better UX. Study 2 aimed to extend the ecological validity of the results of Study 1 by implementing a visual search on multiple screens and investigating the impact of similarity grouping on UX.

## Study 2

The goal of Study 2 was to confirm the effect of color guidance and similarity on reaction time in a more realistic context (objective 1) and to analyze the importance of a quick search for soft dimensions like UX and intuitiveness (objective 2). To this extent, we aimed to increase the visual similarity of the search task towards a more realistic one. On this account, the icons were presented on multiple screens. Additionally, we presented each icon with a name indicating the icon’s purpose to simulate the fact that users’ search for an app is always based on a certain goal (e.g. checking for train connections, not for a train icon as such). Hence, the search space was enhanced and users had to swipe to navigate between the different search screens. However, in order to make Study 2 comparable with Study 1, the presentation of the exact target icon before the search display was retained in Study 2. Regarding the efficiency of the search, we expected to find similar results to those obtained in Study 1. Based on the CUE model (Thüring & Mahlke, 2007; Minge et al., 2016), we further expected that this efficiency correlates with UX components. Mainly, it should affect intuitiveness and perceived usability. Additionally, we expected users to notice the more efficient search if there is a pop-out effect of the target.

### Methods

#### Participants

A study was conducted with 36 participants (19 men) with an average age of *M* = 25.1 (*SD* = 3.5) years. The required sample size was based on the balancing procedure. Based on the pwr-package (Champely, 2018), the given sample size of 36 was sufficient to detect effects with a *d* > 0.70 at an *α-level* = 0.05 and a *power* 1-β = 0.8. Thirty-four of the participants reported that they owned a mobile touch device (smartphone and/or tablet) and used it on a daily basis. All participants indicated that they had normal or corrected to normal vision and that they were not color-blind. Participation was voluntary; all participants were either offered course credit or money for participation. All participants gave their informed consent according to the WMA Declaration of Helsinki. Additionally, the ethics committee of the Technische Universität Berlin confirmed that Study 2 showed no critical aspects regarding privacy, anonymity, and other basic features before the study.

#### Stimuli and task

To enhance the applicability of Study 2 to real-life interactions with mobile touch devices, four features of the stimuli were changed compared to Study 1. First, the presentation of the search screen was adjusted to resemble a typical smartphone. The screen showed a picture of a black iPhone 6 in its original size (13.8 × 6.7 cm) in the middle of a white screen. All icons depicted on the smartphone’s white background had a size of 1 × 1 cm with an inter-icon gap of 4 mm as in iOS 10. Participants sat in front of the device, which was lying horizontally on a desk. The second adaption of the first experiment was the experimental task, which was redesigned to further approximate a real-life scenario. In everyday life, icons may be distributed on a number of screens. Thus, when searching through a screen, the icon can either be on the currently visible screen or the user has to swipe in order to look for the icon on a different screen. Therefore, we always presented two screens, each with 24 icons. Navigating between the screens was possible with a swipe movement. The target icon was always present on one of the two screens. The probability of the icon being on the first or second screen was held equal. A further aspect in which this experiment differed from the previous one was the total number of icons. In order to increase the variability of the icons and to be able to use 48 differing icons during each trial, the total number of icons was increased from 26 (Study 1) to 60 (Study 2). Similar to Study 1, all icons were transformed into a flat design and colored with the 20 colors previously described. This resulted in a total of 1200 colored icons (60 icons × 20 colors). Again, overall luminance and percentage of colored area were not controlled. In contrast to Study 1, each icon was given a name (such as “e-mail”, “puzzle”, “local traffic”, “food delivery service”, etc.) to include a dimension of functionality. A pre-study was conducted in which icons with easily distinguishable symbols were matched to application names. During this process, most icons from Study 1 were replaced by new ones.

Summarizing the above-mentioned differences between both experiments, these four changes in stimuli led to a different task compared to Study 1. Owing to the added second screen, participants were confronted with a larger search space (2 × 24 icons), an additional movement was required (swipe), and they also had to be aware which screen was currently visible in order to navigate between the two screens. This awareness was supported by a sign at the bottom of each screen, similar to the “bubbles” used in the Apple iOS, indicating which screen was currently visible. The dimension of app functionality further added to the complexity of the task. Even though participants did not have to learn the names/functions of all icons, the target was always presented with its matching name/functionality. Thus, the representation of the target consisted of both a visual cue and a semantic one.

#### Design and operationalization

The first objective of Study 2 was to replicate the similarity effect on reaction time in a more realistic context than in Study 1. Hence, Study 2 was also based on the factors TDS and DDS. Both factors were manipulated in the same fashion as in Study 1 (see Fig. 2). Set size was held constant at a level of 24 icons per search screen, leading to 48 icons per trial. Target presence was replaced as a factor by the factor target screen (i.e. the presence of the target app on the first or second screen). Although this variation did not originate from a theoretical framework but rather from a real-life scenario, the splitting of the search space into two screens was thought likely to have a systematic effect on reaction times. Therefore, Study 2 consisted of a three-factorial within-design (2 × 2 × 2) with the independent factors TDS, DDS, and target screen and the dependent variable reaction time.

As a second objective, we aimed to analyze the importance of a quick search for soft dimensions such as UX and intuitiveness. To this end, for each of the four similarity conditions (TDS × DDS) we presented a paper–pencil questionnaire with variables measuring intuitiveness, perceived usability, perceived aesthetics, emotions, a global UX evaluation, and passage of time judgments, as well as a short interview with open questions. At the end of the experiment, an overall interview was conducted. All questionnaires and questions were in German.

Intuitiveness and perceived usability were measured using the INTUI (Ullrich & Diefenbach, 2010) which is based on a 7-point semantic differential and consists of five subscales. In this study, only two of these scales were of interest and are therefore presented: intuitiveness (originally “Bauchgefühl” = gut feeling) with four items (e.g. “When I made use of the colors in my search, I acted rationally” to “…I acted spontaneously”) and perceived usability (originally “Mühelosigkeit” = effortlessness of use) with five items (e.g. During using the color scheme of the icons, I reached my goal with effort “…I reached my goal with ease”). Perceived aesthetics was measured with the VisAWI short (Moshagen & Thielsch, 2013), a four-item questionnaire based on a 7-point Likert-scale with the poles “strongly disagree” and “strongly agree” (e.g. “Everything goes together on this site”). The scales for emotions and global UX evaluation were taken from the meCUE (Minge & Riedel, 2013). Emotions consisted of two subscales measured on a 7-point Likert-scale from “strongly disagree” to “strongly agree.” These subscales are positive emotions (e.g. “The color design of the icons makes me feel euphoric”) and negative emotions (e.g. “The color design of the icons makes me tired”). A single item, based on an 11-point semantic differential ranging from “The color design of the icons was bad” to “good,” was used for the global UX evaluation. On top of the UX-related constructs, we also included one item to measure the passage of time, which might reflect the participants’ perception of differences in reaction time. The item was based on a 7-point semantic differential ranging from The time it took me to find an icon was “long” to “…short.” All questionnaires except the item for passage of time are validated German questionnaires.

The interview after each similarity condition was verbally administered. It consisted of the following questions:

- How did you like the color scheme?
- Would you like to have such a color scheme on your smartphone?

The final question was always accompanied with a short description of the similarity condition. In the conditions in which TDS was low, we additionally asked how the individuals would feel about an algorithm which could predict the app the user is searching for and would color this app in order to achieve a pop-out effect of this app during the search. The final interview at the end of the experiment consisted of the following questions:

- Would you like to change the colors of your app icons?
- How would you feel about app icon colors that represent the category the app belongs to, e.g. all gaming apps are red and all communication apps are blue? Would you like to pick the colors of the categories yourself?^3^

#### Apparatus

The study was conducted on a Lenovo think pad with a capacitive touchscreen. The experimental procedure was implemented with the help of the Psychophysics Toolbox Version 3 (Brainard, 1997; Kleiner, Brainard, & Pelli, 2007; Pelli, 1997) in Matlab 9.1 (R2016b).

#### Procedure

Trials started with the question (“Ready?”) displayed in the middle of the screen. Participants were instructed to hold their forefinger over the text and tap the screen when they were ready to start. Once tapped, a colored target icon and its matching name/function were presented for 2 s and were then followed by the search task. Each search task consisted of two screens with 24 colored icons. In accordance with the concept of a “home screen,” the first screen was always presented as default and the second screen could be reached by sliding to the left. Participants could swipe between the two screens as often as they liked. Screen swipes were registered in a log-file. Participants were instructed to search the target icon and to tap it as quickly as possible. After the response, a feedback screen was shown with information regarding the correctness of the response.

The experiment was divided into four blocks according to TDS and DDS. Each block consisted of 30 visual searches and was followed by the above-mentioned questionnaire and the short interview. As previously described, the target appeared an equal number of times on each of the screens. Each icon was shown twice as a target during the whole experiment but never twice in the same block. Moreover, no icon was presented twice during one trial. Similar to Study 1, target color, color groups, and target position were balanced for each participant. At the end of the experiment, a final interview was conducted. In total, the experiment took about 55 min.

#### Data analysis

Excluding set size as a factor meant that slopes over set size could no longer be computed, so a slightly different data analysis strategy was implemented compared to Study 1. Hence, the following analysis is based on reaction time values. However, a shorter reaction time compared to a longer one can also be described as a more efficient search, so we expected to find the same interaction pattern between TDS and DDS on reaction time values as in Study 1. More precisely, we expected an increase in reaction time values as the target grew more similar to the distractors. This effect was expected to be more pronounced when DDS was high compared to low.

Data analysis was conducted with R version 3.3.2 (R Core Team, 2016) and the same packages as Study 1. Similar to Study 1, all incorrectly answered trials (1.6%) and all trials with unnecessary swipes (5.9%) were excluded from the data before the data analysis. Search time was defined as reaction time between the onset of the screen with the target and the correct answer. Again, reaction-time data were transformed using the Box-Cox function (*λ* = − 0.95) to correct for positive skew (Venables & Ripley, 2002). Subsequently, an outlier analysis was conducted to exclude trials with reaction times differing by more than two standard deviations from the mean, calculated separately for each participant and condition.^4^ The remaining data (89.5%) were averaged over the eight conditions (TDS × DDS × target screen) and used for the statistical analysis with the help of an ANOVA.

The questionnaire data were digitized. In accordance with the questionnaire guidelines, predefined items were reversed and mean values were computed for the scales intuitiveness, perceived usability, perceived aesthetics, positive emotions, and negative emotions. Global UX evaluation and passage of time judgments were not transformed as they were single-item scales. Most of these values, however, were not normally distributed over participants. Moreover, variances in the TDS low conditions were very small for the scales perceived usability and passage of time judgments. Overall, the skew of the data was too strong to allow a parametric analysis. Thus, as a robust test for all questionnaire data, we used the R function “wwtrim” from the WRS package (Wilcox & Schönbrodt, 2017). The function calls for a robust test for a two-factorial within-design that gives an estimate of the significance of both main effects and the interaction based on trimmed means (Wilcox, 2012). The percentage of trimmed means was kept at the default value of 20%. This procedure was used to analyze the effect of TDS and DDS on the questionnaire data.

In addition, we also analyzed the correlation between reaction time and the scales of the questionnaire. As TDS and DDS were within-subject factors, each participant had delivered data in four conditions. Bland and Altman (1994, 1995) suggest two different approaches to handle dependencies due to repeated measures in correlations: one could either focus on the overall effect (e.g. do individuals with a high value of reaction time also show low usability ratings?) or focus on the within-subject correlation (e.g. does an increase of reaction time in one participant go along with a decrease in the usability rating of the same participant?). In the first case, the data need to be averaged for each individual before the correlation analysis. In the present dataset, such an averaging would not be very informative because the four data points were collected under very different conditions varying in TDS and DDS. Therefore, we chose to focus on the within-subject correlations. These are especially interesting because the questionnaire data consist of rating scales, which call the participants to compare internally their experience between the different conditions. On this account, we used the rmcorr package (Bakdash & Marusich, 2018) to compute the within-subject correlation coefficient *r* based on the procedure proposed by Bland and Altman (1995).

The qualitative data from the interviews were analyzed regarding keywords describing positive or negative reactions to the interaction. In other words, similar statements were clustered into key statements. Subsequently, the frequency of each key statement in the sample was computed. If a statement was too ambiguous to be assigned in one cluster, it was dropped.

All presented error bars were corrected for within-subject variance by the method suggested by Cousineau (2005). All post-hoc t-tests were corrected by Bonferroni–Holm correction.

### Results

#### Objective 1

We conducted an ANOVA (type III) on the averaged transformed reaction times with the independent within factors TDS, DDS, and target screen. The results are presented in Table 2.

**Table 2:** ANOVA (type III) on averaged values of transformed reaction time

	F	dfs	*p*	ges
Intercept	13.89	1, 35	< 0.001	0.20
TDS	1352.37	1, 35	< 0.001	0.79
DDS	1.92	1, 35	0.174	0.00
Target screen	4.12	1, 35	0.050	0.01
TDS: DDS	98.95	1, 35	< 0.001	0.17
TDS: target screen	65.11	1, 35	< 0.001	0.06
DDS: target screen	0.90	1, 35	0.350	0.00
TDS: DDS: target screen	3.06	1, 35	0.089	0.00

Effects of target–distractor similarity (TDS), distractor–distractor similarity (DDS), and target screen on transformed reaction time. Generalized eta square (ges) was computed as the effect size. Based on Bakeman (2005) a ges of 0.02 can be seen as a small effect, one of 0.13 as a medium effect, and one of 0.26 as a large effect

**Fig. 4:**
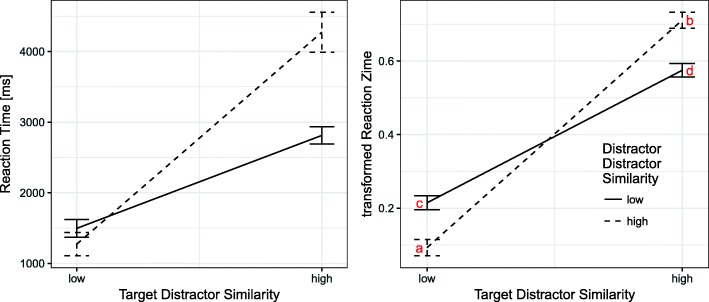
Interactions of TDS and DDS on reaction time and transformed reaction time. Smaller values indicate a quicker and more efficient search. To compare the results with Fig. 1 and Fig. 3, the letters a to d were added to the *graph* on the *right*. All analyses were conducted on transformed reaction times (*right graph*) but original values of reaction time are shown in the *left graph* for easier interpretation

Similar to Study 1, we found a large main effect of TDS indicating that reaction times were much smaller when TDS was low compared to when it was high. Even though there was no main effect of DDS, there was a significant interaction of medium effect size between the two similarity dimensions. The interaction is visualized in Fig. 4. The graph shows that the effect of the TDS condition was stronger when DDS was low (point c to d) compared to when it was high (point a to b). Thus, in line with slope values in Study 1, reaction times were significantly lower with high DDS compared to low DDS when TDS was low (points a and C, respectively), *t*(35) = 8.01, *p* < 0.001, *d* = 0.96. When TDS was high, values were larger with high DDS compared to low DDS (points b resp. d), *t*(35) = − 6.68, *p* < 0.001, *d* = 0.99. A more detailed comparison of the reaction times in Study 1 and Study 2 can be found in the Appendix.

Moreover, we found an interaction between TDS and target screen: the difference between the TDS conditions was more pronounced when the target was on the second screen. That is, when TDS was low, reaction times were slightly higher for targets on the first compared to the second screen, *t*(35) = 3.53, *p* < 0.01, *d* = 0.33. When TDS was high, reaction times were lower for targets on the first compared to the second screen), *t*(35) = − 6.49, *p* < 0.001, *d* = 0.85. Thus, the disadvantage of high TDS was bigger on the second compared to the first screen.

An analysis of the swipe data can be found in the Appendix. We reran the analysis with only those individuals who used a smartphone on a daily basis and the results did not differ from the results described above.

#### Objective 2: Quantitative data

We conducted a robust test for a two-factorial within-design (Wilcox, 2012, for more information see “Data analysis” in the “Methods” section) with the independent factors TDS and DDS for each of the variables of the questionnaire, i.e. intuitiveness, perceived usability, perceived aesthetics, positive and negative emotions, global UX evaluation, and passage of time judgment. The results are presented in Table 3.

**Table 3:** Robust tests with the effects of TDS and DDS on intuitiveness and UX dimensions

Effect of TDS and DDS on	TDS		DDS		TDS:DDS	
	*Q*	*p*	*Q*	*p*	*Q*	*p*
Intuitiveness	65.71	< 0.001	0.44	0.505	22.17	< 0.001
Perceived usability	182.78	< 0.001	11.30	< 0.001	54.91	< 0.001
Perceived aesthetics	11.35	< 0.001	13.47	< 0.001	0.01	0.916
Positive emotions	15.88	< 0.001	2.92	0.088	2.85	0.092
Negative emotions	27.11	< 0.001	19.02	< 0.001	5.34	< 0.05
Global UX evaluation	22.65	< 0.001	25.51	< 0.001	6.94	< 0.01
Passage of time judgment	139.02	< 0.001	13.26	< 0.001	24.00	< 0.001

**Fig. 5:**
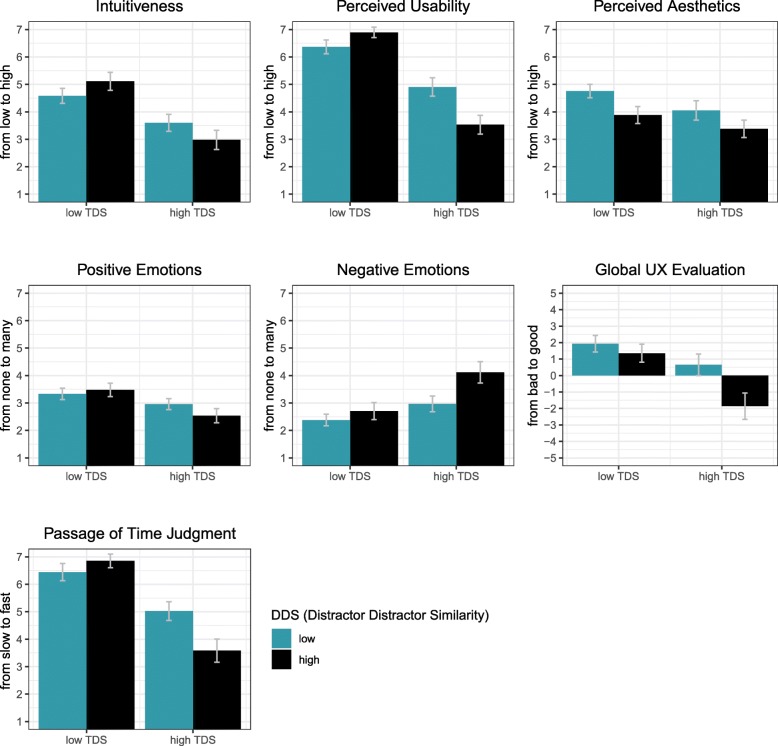
*Bar graphs* of the seven rating dimensions: intuitiveness, perceived usability, perceived aesthetics, emotions, global UX evaluation, and passage of time judgments. On the *y-axis*, we present the range of the scales. The first adjective (from …) stands for low values and the second adjective (to …) stands for high values

As a conclusion, we found a main effect of TDS on all scales indicating that participants experienced low TDS conditions as being more intuitive, more usable, and more visually attractive than high TDS conditions. Moreover, low TDS conditions elicited more positive and less negative emotions and received a higher rating on the global UX evaluation. The passage of time judgment reflected the differences in reaction time as participants perceived the time it took them to find an item as shorter when TDS was low compared to when it was high. However, for most scales there was also an interaction effect between TDS and DDS. The pattern of this interaction was very similar for intuitiveness, perceived usability, positive emotions, and passage of time judgment. In these cases, the most positively rated condition based on visual inspection was always the TDS low and DDS high condition. Compared to the reaction time data, this condition corresponds to the shortest reaction times (point a in Fig. 4). However, this was not the most positively rated condition when looking at perceived aesthetics and global UX evaluation. For these two scales, participants gave their highest rating for the TDS low–DDS low condition. The most negatively rated condition based on visual inspection in all scales was the condition with both TDS and DDS high, which also had the longest reaction times (point b in Fig. 4). These results are presented in Fig. 5.

In addition, we looked at the within-subject correlations (Bland & Altman, 1995) between the reaction time data and the rating scales from the questionnaire. The within-subject correlation coefficient *r* describes how high the interrelationship between two variables is within subjects. The correlations showed that participants rated the interaction to be less intuitive, *r* = − 0.655, *p* < 0.001, and to have a lower usability, *r* = − 0.795, *p* < 0.001, the longer reaction times they experienced. A similar pattern was found for the global UX evaluation and the passage of time judgement: the longer the reaction time grew within individuals, the more negative the overall UX evaluation became, *r* = − 0.655, *p* < 0.001, and the less likely it became to rate the reaction times as being short, *r* = − 0.885, *p* < 0.001. This finding was in line with the correlations between reaction time and emotions, as there was a negative correlation between reaction time and positive emotions, *r* = − 0.468, *p* < 0.001, and a positive correlation between reaction time and negative emotions, *r* = 0.476, *p* < 0.001. The smallest correlation coefficient was found between reaction time and perceived aesthetics, *r* = − 0.312, *p* < 0.001.

#### Objective 2: Qualitative data

The responses from the interviews conducted after each condition supported the above-mentioned ratings. When asked how they liked the color scheme, most participants gave positive responses for the TDS low–DDS low condition. Typical comments, as well as the frequency of positive, neutral, or negative answers after each condition, are presented in Table 4. It seems clear that participants liked TDS low conditions for their usefulness but they found them more boring, especially when DDS was high. Regarding the TDS high–DDS low condition, the results were mixed. However, participants mostly agreed in their negative comments about the TDS high–DDS high condition, calling it “not useful,” “awful,” or “confusing.”

**Table 4:** Subjective evaluations of the similarity conditions with typical comments presented in round edges

Condition	Positive answers	Neutral answers	Negative answers	Total
TDS low–DDS low	22 (useful, convenient, nice for the search, plain, colorful)	11 (useful but ugly colors, sterile, neutral, boring)	2 (too bright, confusing)	35
TDS low–DDS high	15 (useful, easy to find, clear, simple, pleasant)	14 (boring but useful, search is good but colors are ugly, functional but ugly)	5 (too bright, not a nice visual picture)	34
TDS high–DDS low	10 (realistic, nicely messy, attractive, takes more effort)	16 (as usual, neutral, boring, monotonous)	9 (messy, long search, chaotic, unclear)	35
TDS high–DDS high	5 (nicely coherent, easy to recognize the icons)	4 (unexciting, monotonous)	24 (not useful, hard to discriminate between icons, exhausting, awful, confusing)	33

When asked if they would like to have such a color scheme on their smartphone, the answers showed a similar pattern over the similarity condition. When TDS was low most participants reacted positively to the idea, which was independent of the DDS condition. More precisely, 78% of the participants in the TDS low–DDS low condition and 81% in the TDS low–DDS high condition indicated that they would like to have such a color scheme on their smartphone. The rate of positive reactions dropped for TDS high conditions. When TDS was high and DDS was low, 42% reacted positively to this idea. In the TDS high–DDS high condition, none of the participants reacted positively to the idea. Even though many participants had reacted positively to the idea of having a mobile touch device on which the target icon showed low similarity to the other app icons, four of the 36 participants expressed concerns about the TDS–low condition. They feared constant surveillance if their mobile touch device knew which app they were looking for. When confronting all of the participants with the idea of an algorithm that predicts the next click and supports it by creating a pop-out effect, more participants uttered concerns; 66% reacted negatively to the idea of such an algorithm, mainly citing privacy reasons. Only 33% of the participants reacted positively to this idea.

In the final interview, we asked the participants whether they would like to change the colors of the app icons on their own mobile touch devices and how they would feel about color coding of apps, in order to represent certain categories with the help of a common color. Participants who did not own a mobile touch device were excluded from this analysis, leading to a sample of *N* = 34. Regarding the first question, 41% of the participants indicated that they would like to manipulate the colors of the apps on their mobile touch device, while 9% were indecisive, and 50% were opposed to the idea. The latter group mostly attributed its opposition to familiarization with current app icon or brand colors and to a lack of interest in the personalization of colors. Regarding the second question, participants showed a more positive reaction. Of the participants, 65% indicated that they would like a color-coded system on their mobile touch device in which apps of the same categories (e.g. games) share the same color. However, only half of these participants would like to pick the colors of these categories themselves.

### Discussion

The aim of Study 2 was twofold. First, we aimed to show that the effect of similarity between app icons persists when the search task is related closer to a realistic use of mobile touch devices (objective 1). This was realized by visually resembling an iPhone 6, by including different screens which were connected by a swipe and by simulating a use-case of each icon represented by a functional name of the app. Similar to Study 1, results showed that the search became more efficient the fewer icons on the screen shared the same color group. Additionally, the data indicated that the disadvantage of high TDS was even bigger when the target was on the second screen. This effect might be due to more effort on the second screen because participants knew that the target had to be there somewhere or due to fatigue because they already had been searching through one screen (24 icons). Even though the current study cannot discriminate between the two possible explanations, the finding clearly shows how important an efficient search is when searching for an app on a touch device. On touch devices, it is rather common to have two or more possible search screens. Just as portrayed in this study, this means that searching for an icon that is located on a later screen includes negative searches before the positive search. Based on the results of Study 2, we would predict that these searches on connected screens are even more sensible to bad icon design (high TDS) than single screen searches.

Second, we aimed to analyze the importance of an efficient search for UX and intuitiveness (objective 2). The subjective data showed that participants were aware of the differences in efficiency between the conditions (passage of time judgments) and that it affected their UX. More precisely, we found the same effect of similarity conditions on the perception of instrumental qualities of the color scheme (usability) and on the intuitiveness of the interaction. Additionally, high within-subject correlations showed that an increase in search efficiency came with an increase in positive ratings regarding intuitiveness, usability, overall evaluation, and positive emotions. Negative emotions decreased with increasing search efficiency. However, other components of UX revealed a different pattern. Participants emphasized the importance of aesthetic aspects (e.g. visual diversity) by rating the condition with low TDS and low DDS as better and visually more attractive than the condition with the highest search efficiency (low TDS and high DDS). This conclusion is also supported by the small correlation between search efficiency and perceived aesthetics. Overall, the results support the notion that a good UX is not only elicited through high efficiency or high usability but also through aesthetical aspects (e.g. Thüring & Mahlke, 2007).

Another important finding concerns the discrepancy between a general (visual) preference and a specific technical solution. Even though most participants preferred the facilitation by colored icons, more than half of them reacted negatively to a technical solution which would automatically predict the user’s next target icon for visual highlighting. The negative reactions were mainly attributed to privacy issues. In sum, the results of Study 2 show that users prefer a supported search as long as it is not visually too monotonous and respects their need for privacy and control over the device.

Even though Study 2 approximated to a realistic use of mobile touch devices, it was still conducted under controlled laboratory conditions and shares similar limitations.

## Discussion

Mobile touch devices have become an important part of daily life. “There’s an app for that” has become a common phrase, showing how ubiquitous apps have become. While the growing number of apps offers users a wide range of possibilities, it also challenges them to find the desired app from all the others installed on the mobile device within a reasonable amount of time. Focusing on this challenge, we explored in two studies how users can be supported in their visual search for a specific icon by attentional guidance based on universally applicable colors.

Basic research has shown that basic attributes such as color can guide the attention of observers efficiently, even if the search space is complex (e.g. Cave & Wolfe, 1990; Wolfe, Võ, Evans, & Greene, 2011). Moreover, efficient visual search can result from similarity relations (Duncan & Humphreys, 1989). Perceptually similar items (e.g. indicated by similar colors) form groups which can be jointly rejected or accepted in the selection process. In line with the knowledge from basic research, enabling the user to visually group app icons based on their color should facilitate a more efficient search on mobile touch devices. In accordance with basic research findings, our results show that the search was more efficient the fewer icons on the screen shared the same color group. This was clear from the interaction between TDS and DDS. It indicates that grouping of icons occurred and that it facilitated a more efficient search. Also, the findings are in line with previous research showing the influence of color when highlighting important aspects of computer pictograms (Bzostek & Wogalter, 1999; McDougald & Wogalter, 2014) or showing the impact of icon grouping (Brumby & Zhuang, 2015; Niemelä & Saarinen, 2000).

Study 2 incorporated a more realistic scenario by expanding the search space over two screens connected by a swipe. Also Study 2 showed that guiding attention by color similarity enhanced the search efficiency. In addition, the results of Study 2 indicated that these searches on connected screens are even more sensible to bad icon design (high TDS) than single screen searches. In sum, knowledge of basic visual search was successfully transferred to the visual search of mobile touch devices.

In Study 2, we additionally investigated whether these findings correspond to the subjective evaluation. To this extent, UX and intuitiveness of the interaction were examined as a function of the similarity conditions. The subjective data showed that participants detected the facilitation due to the similarity manipulation very accurately and that it increased the perceived instrumental qualities and the perceived intuitiveness of the device. Overall, they showed a preference for a more efficient search but preferred a more diversified visual design, which can be seen as a non-instrumental quality of the device. These results are in line with the CUE model (Thüring & Mahlke, 2007; Minge et al., 2016) which predicts an effect of system characteristics such as efficiency on the perception of instrumental qualities (e.g. usability) and the emergence of an overall UX based not only on the perceived instrumental qualities but also based on the perceived non-instrumental qualities (e.g. aesthetics). Additionally, the qualitative data of Study 2 suggested that users’ needs should not only be considered with regard to an efficient and pleasing interaction but also with regard to privacy and control issues. Even though the idea of an intelligent, adaptive system supporting their search was rather popular in our sample, most participants uttered their concern regarding data acquisition and processing within such a system.

In conclusion, the results showed that search efficiency can be supported via similarity grouping with a simple color manipulation, leaving other design aspects of complex icons aside (e.g. luminance, shade, orientation, forms, and percentage of colored area). Such a color manipulation should be of interest to developers and designers of mobile operating systems, aiming to design for a better UX. To this end, an adaptive color manipulation could be implemented to create pop-out effects. In real-life, the target app constantly changes. Hence, creating a pop-out effect as portrayed here requires the system to know what the user is looking for and to change the display accordingly. A technical solution could be implemented with an algorithm predicting user behavior (based on, e.g., former use, location, time of day, information gathered from other applications). An alternative to an algorithm could be offered by allowing users to categorize their applications and to create a visible indicator for the related category. Up to now, categorization can mainly be done by creating folders, but indicating a category by color might also be of interest to users, as Study 2 showed. Designers could either provide ready-to-use color schemes or leave the categorization and the choice of color to the user. Color-coded categories could make it easier for the user to remember what the target looks like before search and to visualize the target.

Overall, allowing more variability to icon design could both enhance efficiency and further empower users by offering user- and/or system-initiated personalization, as has been shown for other contexts (e.g. Sun, May, & Wang, 2016). Furthermore, it offers great possibilities to meet users’ needs for organization (Böhmer & Krüger, 2013) and for aesthetically pleasing icon design (e.g. Luo & Zhou, 2015). On the one hand, this is interesting for inexperienced users and the elderly. Böhmer and Krüger (2013) found that inexperienced smartphone users organized their apps less than experienced users did. Thus, a simple organization scheme by similarity grouping of colored icons might support inexperienced users. On the other hand, highly experienced users might also benefit from more variability and personalization in icon design. Although the bottom-up guidance of attention by color and grouping should influence experienced and inexperienced users similarly, top-down factors might moderate the guidance differently. Semantic categories build through previous interactions might override the grouping effects of color. In the present studies, only ownership and daily use of a touch device was assessed. As almost all participants owned a smartphone and used it daily, the conclusions based on an analysis with this subset remain the same. A more subtle distinction between different levels of experience might have offered divergent results and could be considered in future studies with special regard to the influence of semantic features.

In addition, color is a feature which is very simple and easy to discriminate and which can easily be perceived, irrespective of language and visual acuity. In this context, negative consequences of similarity grouping should be examined in greater detail. McDougald and Wogalter (2014) have already reported a disadvantage when irrelevant areas are highlighted by colors. Likewise, an uncontrolled similarity of colored app icons (e.g. many blue social media apps such as Twitter, Facebook, Skype, etc. on one screen) could have its cost in the form of a longer search time compared to unique colors, especially for icon-congested screens. The discriminability of colors on different screen types and across diverse user groups (e.g. elderly users) are also important topics to consider in order to ensure a more efficient visual search on mobile touch devices.

### Limitations of present research

Although it was a first approximation to include a second screen to the search task with swiping, an important aspect of touch device use, future work is necessary to further substantiate that colored icons improve this aspect in ecologically valid scenarios. In particular, the importance of the familiarity of app icons and their location was neglected in the present studies. In a real-life setting, the icons are not randomly distributed on a personal device and repeated interaction with the device leads to learning effects. Hence, both the context of target icons and memory of target location play a key role during icon search. In the contextual cueing paradigm, spatial configurations are associated with target position (Chun & Jiang, 1998). Results consistently show that people can rapidly learn numerous target–context associations even with little attention paid to the context. Shi, Zang, Jia, Geyer, and Müller (2013) investigated the contextual cueing effect when icons are rearranged on a touch display. They rotated displays from a landscape to a portrait mode and varied which icons or rather which relationship between icons (i.e. remapping mode) changed with the display rotation. When the positional order (left to right and top to bottom) of the icons in the portrait mode was the same as that in the landscape mode, no contextual cueing gain was found. In contrast, the contextual cueing effect was replicated when only a few icons changed their position with the rotation of the display. The authors concluded that a remapping allowing a good predictability of the target position or retaining the main gist of the display facilitated the gain of contextual cueing. In line with older studies, Shi et al. (2013) also reported that the gain of context takes some time to be deployed. The question remains whether a colored icon arrangement is still beneficial for the icon search when the conditions of a memory-based search are fulfilled (i.e. the location of icons remains constant).

Would the contextual gain remain observable if the search is very efficient (e.g. due to salient target features) or if semantic properties (e.g. the identity of target) are relevant during the search process? Geyer et al. (2010) have demonstrated that contextual cueing facilitates target detection on an early locus of the selection process. They conducted three studies in which they investigated the contextual cueing effect of singleton colored and singleton orientation targets, respectively. The results revealed a robust contextual cueing effect in these pop-out search situations. They concluded: “As a result, a memory-based guidance signal (contextual cueing) may reinforce bottom-up saliency computations, expediting and enhancing target pop-out.” (Geyer et al., 2010, p. 8). In other words, repeated item locations even facilitate visual search if a “pop-out” stimulus guides attention (i.e. efficient search). Consequently, the familiarity of the icon arrangement on mobile touch devices might be also an important factor, even in search conditions which have led to a very efficient search in the present studies (e.g. high TDS).

Concerning semantic features, it is not yet clear to what extent the target-defining information (*what* information, e.g. identity and semantics of the target) modulates the contextual cueing effect (related to the *where* information, Geyer et al., 2010). On the one hand, the spatial domain has often been emphasized in contextual learning (e.g. Olson & Chun, 2002). Consistently, the spatial configurations (*where* information) dominated the semantic properties (*what* information) of items (Jiang & Song, 2005), and the gain of contextual cueing did not depend on identity information (Nabeta, Ono, & Kawahara, 2003). Hence, the benefit of grouping by color similarity might fade in a situation of a fixed icon arrangement on a mobile touch device. On the other hand, semantic properties can facilitate perceptual and mnemonic processes (e.g. Konkle, Brady, Alvarez, & Oliva, 2010). More recently, Makovski (2016) concluded that contextual learning was found only when spatial (*where*) information and semantic (*what*) information were repeated together. He replicated the contextual cueing effect in a visual search task for images of real-world objects. Hence, semantic properties (*what* information) might be beneficial, even if the spatial arrangement (where information) is held constant. One way that semantic features might facilitate visual search on mobile touch devices even if the spatial positions of icons remained constant is the formation of semantic groups. Such semantic groups might comprise icons for sports apps or icons for news apps (similarity by content or categorical effect, e.g. Jonides & Gleitman, 1972; Schmidt & Zelinsky, 2009). The perception of semantically similar items could then be supported by coloring (e.g. Duncan, 1983; Smilek, Dixon, & Merikle, 2006).

In the presented studies, semantic information was only partially included but neither relevant for the search nor for visualizing the target before the search. In both studies, the target was presented visually, which was accompanied by an app name only in Study 2. Thus, even though an additional comparison between the reaction time patterns of Study 1 (semantic information excluded) and Study 2 (semantic information included) revealed very similar patterns for both studies (compare Appendix), there is no conclusion to be drawn about the impact of semantic processes on the relation between color grouping and search efficiency. Moreover, other authors (Brumby & Zhuang, 2015) have already been able to show that a fit between visual and semantic grouping can support search efficiency in human–computer interaction.

In sum, the influence of saliency, semantic features, as well as memory determines the efficiency of target selection in complex search scenes and underline that the investigation of target memory and contextual cueing effects on mobile icon search should be one of the next approximations to a more realistic use case of the present research ideas.

In regard to the present results, colored icons might be beneficial when the arrangement of icons is not yet familiar (e.g. a new device) or when conditions of a memory-based search do not exist (e.g. change of display mode). Further, it might support a memory-based or semantically based search if previous positions or semantic categories were highlighted. Then, similarity grouping might guide the attention rather indirectly. In an ongoing study, we are investigating the effect of fixed target locations and categorical learning on search efficiency and UX of a mobile icon search. The interaction with similarity grouping will also be considered. With regard to individualized app design and UX, the user might choose the mapping of app content and color (e.g. all icons representing sports activities are colored in green). It would also be interesting to transfer similarity grouping results to self-chosen groups and location arrangements.

## Conclusion

A total of 1.9 billion smartphone users in 2015 (Statista, 2016) and billions of app-store downloads (Wikipedia, 2016) reveal a huge diversity of functional add-ons that aim at supporting users in different everyday activities. Quick and easy installation and low prices lead to a steadily increasing availability of apps. Thus, the users’ intention to increase their productivity or to fulfill their (hedonistic) needs through the use of certain apps is faced with a complex and often inefficient visual search task, arising from the increasing number of apps, the mobile use environment, complex motoric responses, and from limited degrees of freedom of icon arrangement. Considering that user-centered app design is incapable of changing the first three of these factors, we tackled the dilemma by optimizing icon design to further support users during their search for a specific app icon. To this extent, we applied prior findings regarding the benefit of attentional guidance by similarity grouping in visual search to the context of mobile touch devices with the help of universally applicable colored icons. Even though typical app icons already make use of color, no phone or software provider has yet presented a color scheme that is consistent across different apps and that supports the visual search for the desired app. Our results show that the efficiency of visual search on mobile touch devices can be modeled by color-based guidance, as predicted by basic research. Thus, color made it possible to group similar stimuli even though all stimuli were as complex as typical app icons. This effect remained significant when participants had to swipe between two screens. The benefit in search efficiency was also reflected in an increased UX. However, the presented studies can only offer the first step when designing for a more supportive search environment. Future studies should consider cognitive processes when imagining the target icon, spatial learning, individual differences in organization preferences, and negative consequences of grouping. Nevertheless, the guidance of attention by color might offer a simple way to enhance interaction with mobile touch devices. Moreover, it can go along with empowering users to personalize their devices to match their hedonistic and pragmatic needs.

## Figures and Tables

**Fig. 6:**
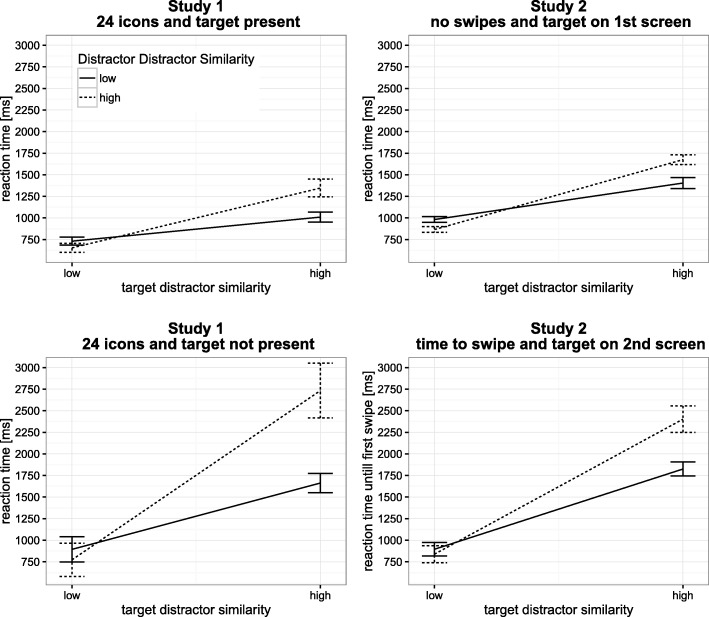
Averaged reaction times of Study 1 and Study 2 in those conditions with 24 icons and a target on the first presented screen (*upper panel*) and in those conditions with 24 icons and the target not present on the first presented screen (*lower panel*)

## Appendix

### Comparison of the reaction time in Study 1 and Study 2

Due to the fact that Study 1 and Study 2 were based on different experimental designs, a comparison between the two studies cannot easily be drawn. While there was only one search screen per trial with one of three different set sizes in Study 1 (8, 16, 24), we presented two search screens (24 icons each) connected with a swipe in Study 2. Additionally, while the target could be either present or absent in Study 1, it was always present on one of the two screens in Study 2. Hence, there are only two meaningful comparisons of reaction time between the two studies. First, we can draw a comparison between the condition “target present – 24 icons” (Study 1) and the condition “target on 1st screen, no swipes” (Study 2) because the requested reaction (to tap the icon) and the set size was the same. Second, we can draw a comparison between reaction time in the condition “target not present – 24 icons” (Study 1) and time until the first swipe in the condition “target on 2nd screen” (Study 2). The latter comparison is also built upon a similar requested reaction (to indicate that the target is not present by either tapping a gray box or a swiping movement) and the same set size. The reaction time in these conditions is presented in Fig. 6.

Figure 6 shows the similar effect of the similarity condition on reaction time in both studies. However, the reactions were slower on average in Study 2 when the target was present. When the target was not present, the reaction times were very similar but the slowest search condition was more pronounced in Study 1. These results could be due to changes in the experimental design, such as smaller icons and an overall smaller display in Study 2, a differing icon set in the two studies, or the additional presentation of a sematic feature (name) during the presentation of the target app in Study 2.

### Analysis of the swipe data of Study 2

In Study 2, participants were confronted with icons distributed between two search screens. They were free to navigate between the two screens by swiping as often as they liked, even though they did not need to swipe at all in half of the trials to find the target icon. In the other half of the trials, one swipe was sufficient to navigate to the target icon. However, participants showed additional and unnecessary swipes in 6.1% of all the correctly answered trials and in 38.2% of the incorrectly answered trials. The unnecessary swipes were especially common in the less efficient conditions. When both TDS and DDS were high, participants performed unnecessary swipes in 13.0% of the correct trials. When TDS was high and DDS was low, the frequency of these swipes was 8.2%. In the TDS low conditions, the percentage of trials with unnecessary swipes was much smaller: 0.2% when DDS was high and 3.0% when DDS was low. Overall, the data indicate that the reaction time increases with each swipe. When using the number of swipes to predict the reaction time of all correctly answered trials in a linear regression, the slope value indicates that the reaction time increases by 2904 ms with each additional swipe, *t* = 68.41, *p* < 0.001. However, this could be an overestimation due to the few data points with very long reaction times and many unnecessary swipes. Repeating the analysis with only those trials with up to two swipes (98.2% of all the correctly answered trials), the slope value indicates that the reaction time increases by 1579 ms with each additional swipe, *t* = 41.21, *p* < 0.001.

## Abbreviations

DDS: Distractor–distractor similarity
ges: Generalized eta-square
TDS: Target–distractor similarity
UX: User experience

## Footnotes

1 Due to the fact that participants were able to move freely while seated during the trials, the distance between the participants’ eyes and the device was variable (approx. 30–50 cm). Therefore, the visual degree of the icons was in the approximate range of 2.8–4.8° in the first study and 1.2–1.9° in the second study.

2 The data analysis of Study 1 was rerun without the outlier analysis, which did not affect the conclusions.

3 There were three additional questions in the interview regarding: (1) the search strategies during the experiment; (2) the organization strategy of apps on the personal mobile device; and (3) the attractiveness of giving apps a colored frame. The results for these questions are not presented in this paper.

4 We reran the data analysis of Study 2 without the outlier analysis. This did not affect the conclusions.
